# Abstract of Proceedings of the Buffalo Medical Association

**Published:** 1867-03

**Authors:** T. M. Johnson

**Affiliations:** Sec’y.


BUFFALO

Medical and Surgical Journal.

VOL. VI. MARCH, 1867. No. 8.

### Original Communications.

ART. I.—*Abstract of Proceedings of the Buffalo Medical Association.*

TUESDAY EVENING, February 5th, 1867.

The Association was called to order at the usual hour by the President.

Members present—Drs. Gould, White, Congar, Camerling, J. R. Lothrop. Strong, Smith, Miner, Trowbridge, Jansen, Wyckoff, Little, Wetmore, Cronyn, Samo, Gleason and Johnson.

Reading the minutes of the last meeting was dispensed with. Under the head of voluntary communications Dr. Augustus Jansen read the following paper, entitled

*A Case of Constipation, complicated with a subsequent discharge of living maggots.*

October 18th, was called to see Mrs. B. W., a poor German woman, aged 40, married, and the mother of six living children. Had not menstruated for the last seven months, yet no signs of pregnancy. She complained of great pain in the womb, and had very severe abdominal singultus, was very restless and excited. I thought it was hysteria, and gave her one-fourth grain of morphia every three hours She took four such doses, and on the 19th she felt a great deal better, complaining only of giddiness and nausea.

Oct. 20th. Said her bowels were costive. I ordered an ounce and a half of castor oil on some hot coffee. She vomited some of it, and it did not move her bowels.

Oct. 21st. Same abdominal spasms, womb felt hard and painful, not unlike a contracted uterus after parturition. Prescribed mass. pill galbani co. gr. x, every two or three hours; took ten doses of it with apparent great relief for the hysterial symptoms.

Oct. 22d. Not having had any stool, I gave her three compound cathartic pills in the morning, and same dose in the evening.

Oct. 23d. No operation. Ordered an enema of two ounces castor oil in a quart of soap-suds, to be repeated three times a day if necessary.

Oct. 24th. Calomel, grs. xv, rheubarb grs. xx, pulv. opi. grs. iij, made into 12 powders; one every two hours; took the whole without effect.

Oct. 25th. Enema of castor oil and soap-suds as often as they came away, unless the bowels should move.

Oct. 26th. Gave her myself an enema with salt water, and it coming away without any cathartic show, I ordered the following: podyphylum gr. xii, ext. hyoscyami gr. vi, div. into twelve pills, one every three hours. Took the whole without avail.

Oct. 27th, 28th and 29th. Countenance and pulse remaining good, I only ordered the same injections, which brought nothing away.

Oct. 30th. ℞ Olei. tiglii crotonis, gtt. xxv, cons. rose gs., make into ten pills, one every four hours, and about a drachm more by inunction over abdomen.

Nov. 1st and 2d. Has taken all the pills, but it did not start the bowels. Continued the injections. Pulse 85.

Nov. 3d. Swelling and redness over neck and face.

Nov. 4th. Croton oil vesication over face. Discontinued internal medications by mouth, but continued injections, and introduced a rectum tube and had her head lowered and hips elevated in order to have the injection reach as far up as possible.

Nov. 7th. Great pain and restlessness, and gave her half a grain of morphia; complains of swelling and scalding in the pudenda.

Nov. 8th. Saw her with Dr. J. R. Lothrop. We could feel distinctly the empacted colon and cœcum. We concluded to try elaterium gr. i, divided in eight doses. She first took $\frac{1}{8}$, secondly $\frac{1}{4}$, thirdly $\frac{3}{8}$, and last $\frac{1}{2}$. With the exception of some nausea she felt no effects of it.

Nov. 9th. A slight show of menstruation set in and she feels comparatively easy.

Nov. 10th. I made her an infusion of half an ounce of socot. aloes in ten ounces of water, simmer down to eight ounces, gave one-fourth of it in a pint of water as an enema. This caused her great tormina, but came off as it went. The infusion of aloes was all used by injections; but it only aggravated her sufferings.

Nov. 11th. Thinking that the elaterium might have been inert, I bought another sample, of which I gave one-eighth of a grain to a middle sized dog, and it worked him rather severely. The patient took one grain of it in two doses.

Nov. 12th. Elaterium had made her vomit some, and she feels a little better. Put her in a warm bath, and after that applied fomentations to her bowels, and prescribed calomel gr. viij, opii gr. ss. She took six of these powders without any effect whatever.

Nov. 13th. At the suggestion of Dr. Lothrop I got a vesic fellis-bovini, the contents of which I diluted with a quart of tepid water, and injected it as high up as possible. This partially relieved the poor woman, and I thought the trouble would soon be over, but the case here assumed quite another aspect, and the battle was to be fought over again. The alvine dejection that followed the ox gall injection was partially liquid, with some very hard lumps, together with an astonishing quantity of living maggots, about half an inch long. I could hardly believe my own eyes, and I felt vividly the corruptibility of our flesh, and how truly the holy man Job, had said, "Putridini dixi pater meas es et soror mea ver-' mibus!"

Nov. 14th. She complained of pain in her bowels, and pointed out to me with her hand the whole track of the ascending, transverse and descending colon, which I found distended, but not quite so hard as before. I ordered another enema of ox gall, diluted with water. This was in the morning, and I did not see her in the evening.

Nov. 15th. Was about the same, but I heard to my disappointment that she had not taken the injection, and ordered it again, besides, gave her eight grains of calomel with half grain of opium. This again brought away a small quantity of hard fæcal matter, literally crawling with worms.

Nov. 16th. Found her with an erythematous eruption over her face, and she had passed early in the morning a very compact lump of white stercoraceous substance, equally swarming with maggots. I gave her another enema with ox gall, and as there was not the slightest sign of mercurialization I ventured another eight grain dose of submurias. hydr. She felt cheerful, but objects strongly to the ox gall injection, as it gives her, she says, "*more tormina and uneasiness than all the other stuff together.*"

Nov. 17th. Nothing had passed her bowels. I found her sitting up in bed and crying; the maggots dropping or skipping out of her nostrils. I carefully examined her mouth, pharynx and nostrils, but I found these parts perfectly healthy. Evidently the maggots must have come from her stomach. She now was not only willing, but very anxious to have the ox gall injections repeated. She took four of them during that day, and towards evening she took infus. senna fol. ℥i, magn. sulph. ℥iij, steeped in ten ounces of water; took the whole; no avail.

Nov. 18th, 6 P. M. ℞ Sp. terebinth. ℥iss, with vit. ov. ij. I prepared it myself; put some coffee with it. She drank it all at once from my own hands. In leaving her I told her that, if she had any stools, to keep at least the first one until I saw her. The following morning being called out of town I did not see her until 5 o'clock P. M. The oil of turpentine had produced the intended effect, both as a cathartic and as an anthelmintic, for it brought off a living, crawling mass of maggots, not unlike a nest of caterpillars recently hatched out. Here are the specimens. The poor sufferer had the same night two more stools, vomited three times, and slept soundly thereafter.

Nov. 20. Painful micturition, caused no doubt by the turpentine, but she was cheerful.

Nov. 21st. Ravenous appetite. Worms skipping out of her nose.

Nov. 22d. Bowels had not moved since 19th; give her an injection with two ounces turpentine in some milk and water.

Nov. 23d. Complaining of dizziness and pain in her head. Prescribed hyd. sub mur. and jalap āā 20 grains, as nothing could induce her to take turpentine again.

Nov. 24th. The medicine had not operated, and I ordered three drops of croton oil to be taken in a soft, stewed prune, every three hours. She took eighteen drops of it without effect.

Nov. 25th. Worms continually dropping out of her nose. I gave her *nolens volens* three ounces of turpentine, in an enema. This produced nothing but a little nausea.

Nov. 26th. Did not see her.

Nov. 27th. More and more worms. Prescribed tart. emet. gr. ij, syrupi scillæ ℥iij, vini ipecac ℥ij, aqua camph. ℥i, a table spoonful every ten or fifteen minutes. Took every drop of it, and vomited three times an abundance of maggots.

Nov. 28th. Ravenous appetite—gave nothing.

Nov. 29th. No stool yet. Prescribed elaterium gr. ss. and four hours after the same. It did not affect her any more than if she had taken so much sugar.

Nov. 30th. Worms crawling out of her mouth and nose, the patient crying bitterly. She had about half a teaspoonful of them before her on the bed. I really was mortified myself and felt for the woman's sufferings, and the more so as she so implicitly and solely trusted in me. After considering the case over in all its bearings, I resolved to make an experiment. I remembered that, when a child, they often made me take a nostrum called Harlem oil, to kill worms, and as that article resembled to my recollection, so much Petroleum, I made up my mind to try the latter. I got some crude Petroleum and gave her at once half an ounce of it. Three hours afterwards she took the same quantity, and after the third dose she had a free and very copious evacuation, in which were myriads of maggots, apparently all lifeless. Her bowels moved every day by taking a drachm of the oil every morning, without any more maggots. Her stools, however, were of clay color.

Dec. 10th. Gave up taking the oil, and I prescribed infus. calumba with tinct. ferri mur. as a tonic. On the 16th the motion of the bowels stopped again, and being again a week without a motion of her bowels, I advised her to take the oil again in teaspoonful doses. She did so, but it did not help her. I gave her then and there a drachm of calomel with a drachm of jalap in three doses, four hours apart, and waited in vain three days for its operation.

On the 21st I was sent for in haste and found her in hysterical convulsions, and unable to pass her water, and the same erysipelatous swelling over neck and face. . Drew off thirteen ounces of highly colored and offensive urine, and gave her at once three ounces of Petroleum, to be repeated every three hours. After the second dose she had a free passage of her bowels, *natural color*, and on the 1st of January was up and about. On the 16th of January she came to my office, and with the exception of suppressed menstruation was doing well. She asked me if I could possibly account for the presence of so many worms. I told her that she might have eaten some tainted meat, and thus have the maggots developed from some larva or fly-blows, as they are commonly called. She said that, although living on very poor diet, and got sometimes cold victuals from public houses, she was very particular in cleanliness, and could not be made to believe that this was the case with her. Now if this was not the cause of this appalling quantity of maggots, what was it? Were they spontaneously engendered in and by the constipated bowels, or were they the cause thereof? There was no biliary secretion in the stools, and whether this had anything to do with it I am not able to say, but thinking that there was no bile secreted, I gave the repeated doses of calomel, and the freer as she never experienced the least inconvenience from it; but whether the calomel had the intended effect or whether the secretion of bile was solely due to the action of the Petroleum, I cannot determine. One thing is certain, Petroleum is a very powerful and safe cathartic, as it proved to be in this case, and also in the case of Mrs. B. living in the same house, who took half a teaspoonful for costiveness, which was removed thereby without any tormina whatever. In extraordinary cases I would not hesitate a moment to give it again. Given in injections it may also prove to be a mild and very efficacious vermifuge.

Dr. Lothrop said that he saw the case with Dr. Jansen when the constipation had existed about twenty-five days. Upon examination the impacted colon could be traced from the head of the cœcum, where there was a large and hard mass, along the ascending and transverse colon to the point where it began to descend. No accumulation could be detected in the descending colon. The obstruction appeared to be at the termination of the transverse colon. But in order that it might be ascertained, whether or not a malposition of the uterus existed, which would act as a mechanical obstruction, an examination was made. The uterus was found normal as to size and position, and freely moveable. As the condition of the woman was good, it seemed to him proper to make a further trial of active cathartics.

One point of interest in the case related by Dr. Jansen was the eruption upon the face, caused by the absorption of croton oil. Before seeing the patient he was inclined to believe that it might have been caused by the contact of the oil with the skin of the face, being conveyed thither by the fingers, as some of it had been applied externally upon the abdomen. But upon examination he was convinced it could not have been caused in that way; as traces of the eruption were found not only over the whole face, but even behind the ears and in the hair. It had the appearance, familiar enough, of the drying up of pustules, such as follows the application of croton oil. It appeared very plain that it was caused by absorption of the oil. The case was not wholly without parallel. Pereira mentions a case where its use was followed by the characteristic pustules upon the face.

The second point of interest was the development of such numbers of living maggots in the human body. In some of its features the case was, as far as he knew, without parallel. He had, however, read the account of a case in which large numbers of maggots escaped from the nasal cavity and mouth. In that case a man was attacked with great pain in the head, followed by swelling of the face, and much general febrile disturbance. In a short time, maggots in great numbers were discharged from the nose and mouth. Means being taken to destroy them, the trouble was soon over. The origin of the worms in such a situation was thus accounted for. The man stated that a few days before, he had handled the intestines of a cow, which he remembered were fly-blown at the time. The ova must then have been inhaled and lodged in the nasal cavity, where they became developed and were afterwards discharged. In the case of Dr. Jansen the worms were discharged by the rectum as well as by the mouth and nose. The ova then must have been swallowed and undergone development somewhere in the intestinal tract, i. e. they must have been introduced from without, as he supposed no one of the medical gentlemen present was ready to believe in *generatio equivoca.* Strange as it might seem, it was possible for the ova to escape destruction and become developed. Whether constipation was due to this or not, certainly, by preventing the escape of the ova from the body it would give opportunity for the completion of the process. Had the bowels been free the ova might have been discharged from the body.

In regard to the fact that the worms were actually maggots, he thought that an examination would satisfy any one. It was certain that they did not resemble any parasite known to inhabit the human body.

Under the head of dissertations upon designated subjects, Dr. Miner read the following upon

*Surgical Remedies — Vesicants, Rubefacients, Setons, Issues, etc., etc.*

Upon your earnest and unanimous invitation, I have reluctantly consented to read a paper, designed as the re-inauguration of a plan in our proceedings, which, if heartily seconded by all the members of the Association, cannot fail to increase the value of our transactions, both to ourselves and others. Happy indeed should I have been, had this task fallen to other and more capable hands, but as you would accept no apology, nor listen to excuse, I have selected a topic as familiar and practical as possible, and ask your indulgence if I express opinions or sentiments which some of you, and the profession generally, are not ready to accept. You will not be asked to change your opinions to suit any new theories or your practices to conform to new modes of reasoning; it is designed only to call your attention to some points of practical importance, believing that errors may thus be avoided and improvements made. This paper has not been written to gain the compliment of your unanimous approval; you are at liberty to differ from the author in your views and to express your opinions. If he should hereafter return such favor, please set it to a just account, and never carry it over to the distant, dearly bought, unhappy and untruthful reconing of personal disagreement. He has sometimes hesitated to speak plainly, fearing to be misunderstood, and presumes others have suffered the same embarrassments.

Vesicants, rubefacients, setons, issues, etc., etc., have been, and still are, employed to a very great extent with the view of producing a derivative or revulsive influence—of inducing an irritation or discharge in one organ which is healthy, for the relief of another which is diseased. Irritant medicines are used chiefly to relieve congestion or inflammation, to modify the undue fullness of the vessels and to relieve pain, the objects for which such remedies are used, are said to be four-fold; to substitute a mild inflammation for an intractable one, to stimulate the absorbants, to act as derivative, and to stimulate the whole system. Inflammation consisting essentially of unusual afflux of blood and stagnation in the over-distended vessels, it is generally intended to modify this tendency in the blood current, and set up a counter current in another direction, for the relief of the first.

The question, in what manner an artificial congestion or inflammation, modifies one which is of a diseased nature, has never been answered with any reasonable satisfaction. Undoubtedly the blood accumulates in the part newly inflamed or irritated, but this fact does not prove a relief or modification of the original diseased action. Recently it is attempted to explain it through the laws of the nervous system. Nervous action is said "to never be equally intense in two distant places at the same time," and that "through these nervous relations, all stimuli, whether by a direct, or indirect action, excite contraction of the blood vessels, and thus produce resolution of engorgements." Formerly, much more than at the present time, irritants, "permanent irritants," as they are called, were held in estimation as remedial means; they were used to evacuate from the system the "precant humors" supposed to circulate in the blood and constitute the essence of disease. When views changed as the nature of disease—when its pathology came to be more carefully studied and thoroughly understood, and when bleeding, antimony, mercury and other remedies of the same class were discarded, or used much less frequently, it was to have been expected that these irritant measures would also have disappeared from the list of common remedies, but this expectation has been disappointed, and blisters, setons and issues still appear as too frequent modes of torture for the invalid.

In support of this doctrine and plan of treatment, it has been shown how much health depends upon the elimination of various deleterious, because effete substances from the blood, through the excretory organs; but it has never been shown that bile, urea, or any other deleterious substance, any such excretion or purification of the blood, can be produced through artificial exutories; nothing but the common products of inflammation can be thus obtained. At the present time the application of these remedies is confined chiefly to the relief of pain, and the modification of inflammatory action, and with a view to these objects their employment is not infrequent. That confidence is placed in these measures by well informed practitioners, and experience quoted as abundantly sustaining belief in their efficiency, is undoubted. Hunter speculated upon their modes of action and was evidently dissatisfied with his best explanations. Authors, have almost without exception, favored the employment of these substances as powerful and valuable therapeutical agents, and if able to explain their action or not, have at least recognized their value as remedial means. It is common for authors to speak of the dangers of suspending these suppurating sores, from fear of cerebral disorders. They say: "No sooner is the revulsive and depletory action of the remedy suspended, than congestion of the brain begins to take place, and unless active measures are taken to avert it, apoplexy is pretty certain to result." Every one is sufficiently familiar with the prevailing doctrines concerning these irritant remedies, and their consideration may be omitted. Common forms of inflammatory disease have been watched and practical lessons drawn from their phenomena. Rheumatic or gouty inflammations which involve the ligamentous tissues of joints, generally limiting itself to the tissue which it invades, is still very apt to extend to the pericardeum, and sometimes to the membranes of the brain; so also mumps—inflammation of the parotid, may involve the testes and mammæ.—The idea is prevalent that there is a translation of the disease from one part to another, an actual endosmosis, and hence the application of vesicants or rubefacients to the primary seat of the affection, from which disease has taken its departure. Without turning aside to consider the phenomena of such diseases at too great length, it will be sufficient to say, that, strange enough, the same theapeutical measures, (counter-irritants) are applied both for the removal of such primary disease, and also to bring it back to its original seat after it has vacated it, both as a cause and cure. The actual phenomena of these diseases are perhaps explainable, but the treatment by vesication or counter-irritation is probably not greatly in harmony with any very correct pathological view.

Blistering is a favorite remedy with many practitioners, and is resorted to for various purposes. Not long since all internal inflammations were treated by external vesication, perhaps solely from its supposed derivative or substitutive effect. Puerperal inflammation once combated with venesection, mercurialization and extensive external vesication, is now more successfully treated with anodynes, and simple fomentations. Inflammation of the lungs and pleura formerly treated by the whole armament of depletion, is now in the best manner controlled by rest, position, and anodynes, the blistering, bleeding and starvation wholly omitted. Inflammation of the eye, in its various forms, formerly submitted to leeching, blistering, cauterizing, low diet, etc., is not often at the present time subjected to the action of such remedies, but terminates more safely under milder measures. The diseases most eminently requiring, as supposed, the action of these remedies, are now known to recover as rapidly and certainly without them, and thus we are furnished with presumptive evidence, at least, that they are nearly, perhaps wholly incapable of good, better, if we could also add of harm, but we are obliged to confess that if unproductive of good, they are yet potent for evil.

We all rely largely upon our own experience in these matters, though experience establishes error more frequently than truth, as the whole history of medicine will show. The experience of physicians from the creation of the world until the present time, appears to favor a general belief in the efficacy of this class of remedies, though there is no doubt but the recent revolutions of practice, have turned out in some measure the constant use of them.

It is remarkable how distinctly we can see whatever we attentively look for and confidently expect to see, and especially so, if those we most respect think they can see the same thing. For the first ten years of my professional life, I could see the great efficacy of blisters, could see the value of setons and the danger of apoplexy when suppurating sores were allowed to heal; could see everything that any one could in this direction. For the last ten years, I am frank to confess, I have been unable to see it; have not seen apoplexy follow the removal of a seton, and am also certain that I never saw it, reasonably traceable to it as the cause; have not seen cerebral congestion after removal of suppurating sores, even of years' standing, but on the contrary observe increase of flesh and strength after amputation for suppurating disease of joints or other structures, and look upon it as proof of returning health. Every few days brings to notice cases of spinal curvature, scrofulous inflammation of joints, or other similar disease, under treatment by blisters, moxa, seton or issue, all of which appears to me now as only adding to the unfortunate victim another and worse curse. They irritate and distress, cause loss of appetite, sleep, and rest, depress the strength and spirits, and are the relics of a vague and exploded theory, which should be buried with the "peccant humors" of antiquity. Strange as it may appear, blisters are still fashionable appliances for the relief of all pains, from undetermined causes; the abdomen, in women, who, after confinement, complain of tenderness, especially if associated with febrile action, is very often found covered with a large blister—blistering is still, with many physicians, a standard remedy in puerperal inflammation. Inflammations of the lungs and pleura, even in children, are often subjected to this unnatural, unnecessary and unscientific treatment; and I may add, that looking over the list of diseases, in which this system is even now strongly recommended in our recent standard works, I find few, if any, in the whole range of known diseases but are thought by some good authority, to be benefited in some of its stages, by this system of practice. Driven from the unnecessary and indiscriminate use of the lancet, from the extravagant employment of depressing arterial sedatives, and some of the more objectionable "heroic remedies" the remaining irritant adjuvants, in the same system, have been retained, and their application scarcely lessened in frequency, excepting by a few who nearly discard them altogether. That there is a popular belief in the curative virtues of the irritant remedies is certain, and it is not uncommon to hear this fact referred to, as apology for their application, "My patient would think I was doing nothing, if I did not apply the blister." On the other hand there can be no doubt but this system of practice, in connection with the unnecessary and useless administration of unpalatable drugs, has had a tendency to drive people from confidence in the practice of the regular profession, to the adoption of some inert, inoperative, unscientific and absurd system, which is less unpleasant in its measures of treatment. We look in all directions but the right one for explanation of the readiness with which the public adopt extravagant, untruthful, useless and inconsistant systems of medical practice; possibly it has not "erred without a cause,"—has adopted them as justifiable measures of self-defense.

Blisters may be applied many times without great pain, possibly even with benefit, for it is not certain, they, and the other remedies of the same class, are never useful; certainly during their action other pains are forgotten—the mind is diverted from the pain of the diseased part, by the more active irritation in the healthy organ. If we had no other and better means of allaying or causing pain to be forgotten, this plea would have some force, but no such circumstances have ever appeared in its favor. The expedient of producing greater pain, for the purpose of diverting attention to a lesser one, perhaps of longer standing, has little to recommend it to favor, nothing since the discovery of the present known agents for its relief. If the author had invented such an accusation, alleging that this class of remedies, is useful, if at all, or mainly so, by diverting attention to another and greater pain than that of disease, he would have been called skeptic, and unbelieving, without faith in the value of medicines generally, and ready to deny or underrate their curative virtues; but this explanation is given by those who advocate their employment, and are most decided in their favor—it is the compliment of a friend and not the sarcasm of an enemy. But it will be said, blisters are useful in diverting the current of blood to other and distant parts, or by its stimulant action, causing contraction in the vessels and consequent relief of congestion and incipient inflammation.—Experiment shows that the quantity of blood in the vessels is not easily diminished, that they contain about the same volume even when large vessels are opened and hemorrhage is considerable; that if the amount is diminished at all, it is only temporarily, since the vessels contain at all times, except in cholera and some other similar disease, about the same quantity, even if the quality is impoverished. That the mind has some influence upon the circulation is not doubted, but that the value of counter-irritation can be established upon any mental control which can be brought to bear upon the circulation, is improbable.

It is not proposed to wholly discard this class of remedies, or to intimate that they are *never* useful; it is rather to call attention to the impropriety of their common and indiscriminate employment. It cannot be doubted that these measures, as remedial means, are gradually disappearing from the list of remedies, and probably the more rapid this change the better, both for the profession and public. That they have any influence for good, at all to be compared with the pain, irritation, distress and discomfort they produce, is no way certain, and when they are almost wholly discarded, physicians will have lost but little from their available means of curing disease. It has been intimated that authors generally favor the employment of these remedies, but it will not be inferred that the opinions expressed as to their abuses, and misuses, are unsustained by the most distinguished observers.

M. Louis says, "Blisters ought to be banished from the treatment of the typhoid affection. If they exercised any influence upon the duration of the disease, in the patients who have recovered, it was by *prolonging* it a little." Again, "I have not only rejected vesication from the treatment of pneumonitis; I have also ceased to employ it in pleurisy and pericarditis. How can we believe that the effect of a blister is to check the inflammation, when this blister is one inflammation superadded to another? in thoracic inflammations their usefulness is neither strictly demonstrated, *nor even probable.*"

"One thing is most assuredly beyond question, and we should never be weary of repeating it, *that the therapeutic value of blisters is unknown;* that it must be studied by the aid of numerous and carefully noted facts, *just as if nothing at all were known about it.*"

There are many theories and speculations concerning these subjects which have not even been mentioned, as it was no part of the purpose to weary you with arguments for or against their use. It will be said that experience sustains the opinion that this class of remedies is powerful and important, and few physicians but will revert back to a case where some of these measures were adopted with unmistakable advantage. We must all guard ourselves lest we adopt opinions upon insufficient grounds. Practical medicine has been retarded more manifestly by this one error, than by any other; and it is a reflection upon the mental acumen of men, if they habitually allow themselves to be convinced without adequate evidence. On the other hand, a readiness to concede what is shown with reasonable certainty to be true, is highly commendable, and I would be the last to underrate the curative virtues of valuable remedies, and the very first, to rid the profession and public, of undue confidence in the properties and uses of all remedies, which are retained from respect to long usage, and the opinions of others, as liable as ourselves to be deceived.

Dr. Congar said, that while he disliked to differ with Dr. Miner in regard to counter-irritation, or any other medical subject, still he must be allowed to dissent from some of the positions taken in this Essay; he could not endorse all that had been stated of the inefficiency of blisters, etc., for the relief and cure of disease; for, after abating the effects of their misapplication in time, place and condition, by the ignorant, the inexperienced, the careless and the hasty, there remained a very large and predominant amount of good from their use in the hands of the profession, if he could judge from an experience of over twenty-five years. Early in his professional life the principle of their employment had been ineffacably impressed upon the memory; and by always limiting their application to an open state of the bowels, and to the commencement of the return of the secretional functions, as evidenced by a perspirable skin in pleurisy, peritonitis, etc., etc., blisters, etc., especially when aided by opium and its preparations, had invariably fulfilled legitimate expectations of relief and cure.

This, he continued, brought to mind a case at the Hospital of the Sisters of Charity some seventeen months ago, which so happily illustrated the principles of application, and the advantages of the appropriate use of blisters, that he was constrained to give a succinct statement of it. It was that of a servant girl, nineteen years of age, in whom the menses had appeared at irregular periods, since suppression some two years previously; the bowels were habitually constipated, except an occasional interruption from profuse diarrhœa; the tongue was red and dry; the abdomen was enlarged to about the size produced by the fifth month of pregnancy; when the lower extremities were drawn up, beneath the integuments, hardness and tenderness were everywhere found; at one time this abdominal hardness had the exact feel of an enlarged uterus of five or six months' pregnancy; and at another, this feel could not be discerned, but in its place, smaller hardnesses, or tumors; and then again, the hardness would be uniform over the abdomen; these, as he learned from his predecessor at the hospital, had been the patient's symptoms since her entrance some weeks before. The bowels had at this time become somewhat more regular in their movements, and the menses were flowing; during the following two weeks no alteration in the course pursued by the retiring physician was made; the pulse ran from ninety-six to one hundred and twenty; the tongue was dark, dry and rough in the centre, and its sides covered with a yellowish brown fur, the teeth showed a collection of sordes, the breath was offensive, and the skin dry. Now, the necessities of the case demanded a change of the treatment; the bowels had not been open for some three days; he commenced by giving castor oil to open the bowels freely, following it by ten grains of Dover's powder, and when from its effect the skin began to perspire, he ordered a blister to cover the whole abdomen, dress with poultices, followed by simple cerate till healed; and also tinct. cinch comp. ʒi, at meals, with plain diet. This was repeated about every fifth day for six weeks: the improvement was very marked immediately after every blister; if the interval was prolonged after the healing of one before the application of another blister, from the time of healing until another vesication, *the patient did not gain;* but while their repetition was made, as fast as the abdominal surface would allow, the decrease of the tenderness and hardness was steady and rapid, the pulse lessened in frequency, the dry, rough, and dark tongue, slowly became moist, smooth, and lighter in color, the offensive ness of the breath, and the sordes on the teeth ceased to be noticed, until at the end of two months the menses returned healthfully, and the patient was in health.

The cure of this case was evidently brought about by the continuous repetition of blisters, applied as he always used them in the conditions named above. These conditions he believed were, in order to be remedial, required by all counter-irritants; consequently, it would be simply impossible for him to concur entirely with the able author of this (in most respects) excellent essay, with which the Society had been favored.

Dr. Strong said, it seems to me, Mr. President, to have been very properly said by the gentleman who preceded me, that the paper which introduces the discussion of the evening, by Dr. Miner, is quite too sweeping and undiscriminating in the positions it takes. And here a word as to the designation of the discussion, viz: "*Surgical Remedies.*" I came in expecting, from the Secretary's note, a discussion upon remedies in surgery. But the paper of the evening seems, if I rightly apprehend it, devoted to impairing our confidence in the whole system of counter-irritation and derivation by blisters, sētons, issues, moxas, etc., etc., in the treatment of all forms and phases of disease. I fail to see the propriety of calling these *surgical remedies*, in any fair acceptation of the term. It seems to me a misnomer. And I was misled by it into supposing that our discussion was to be on the merits of our resources in surgical practice. But thus much only as apology for lack of preparation for the discussion.

The assault is made upon blisters, etc., in the treatment of disease. For although the text of the paper contends only for their being *almost* wholly useless, or worse than that, I think the inference is not very wide of the mark, that the author *wholly* discards them. Now I am very far from contending or believing that they have not often been, or are not liable to be used injudiciously and unnecessarily—especially when prescribed for infants and very young children. In such cases, if used at all, they (I mean now blisters) should be used with the utmost caution. But to argue from their abuse or their indiscreet use, that they are *never* of service, and should be wholly ignored, it strikes me is a strange *non sequitur.*

I would like to be told what medicine we have, or what resource we have of any potency either in medicine or surgery that is not obnoxious to the same objection. Of course this mode of argument is hardly worthy a place among men of sense and discrimination. If I understand the purport of the paper, it deduces from the fact that authorities differ as to the *rationale* of their effect, one claiming that it is through the nervous system, another thro' their effect in determination of blood, or upon the capillary circulation, others in still other ways—that hence they are unworthy to be held to or retained.

Now I have long since ceased to spend much time or breath upon the *modus operandi* of medication, whether external or internal. But what can be said in this regard against blisters, etc., etc., that cannot be alleged with equal truth against every medicine or means to which we resort? Is it fair to claim that because we do not fully understand their mode of operation, we should shut our eyes to their palpably beneficial effect in modifying and abating morbid processes? The truth is, and it may as well be admitted, that the *modus operandi* of medicaments, external or internal, has ever been, is yet, and perhaps may ever remain, almost wholly a *terra incognita.* But this style of logic is too rudimentary to waste words upon.

Again, the paper says, in substance, that the pain they cause is as bad as, or worse than, the pain, to relieve which they are prescribed. This objection is weak, because, 1st. They are rarely, if ever, prescribed with primary intention of relieving pain. They relieve pain only when, and to the extent that they reduce and obviate inflammation. Moreover, it has always appeared to me that the alleged pain from a blister is wildly exaggerated. It is very rarely, under my observation, that pain is complained of from a blister, except sometimes at the moment of drawing, or elevation of cuticle. But even if it were, is the pain of a surgical operation for the removal of a non-painful disease a valid objection to its being resorted to? Where will this kind of argument lead us? But this style of objection, too, seems concededly frivolous; and the true appeal is taken, that, viz: to results of experience and observation. And here, if I understand the paper, the Doctor takes the bull squarely by the horns, and denies that the results of observation and experience do or can sustain the practice. The paper before us cavils at the results of observation as unreliable, and as likely to lead us astray as to guide us aright. Candor compels the admission that they many times have been so. Subsidence of disease often proving coincident with, rather than standing in the relation of effect to cause, to the means and measures resorted to. But after all abatements and concessions from imperfect observations, I still must contend that carefully made, sufficiently multiplied, bed-side observations and comparisons, are our main, if not only reliance, in deciding upon the merits of remedial resources. Now the effect of the epispastic, the moxa, etc., may not be quite so palpable as amputation at the hip-joint, but I think nearly all of the best *medical* observers of the profession regard their beneficial effect about as demonstrable; I mean as demonstrable to any but a purely *surgical* mind. They [surgeons, pure and simple,] seem to constitute a class *sui generis*. Living and moving in the sphere of the palpable, the tangible, the mechanical—anything that is not removable by the *knife*, is not amenable to treatment. If it cannot be cut out, or cut off, it is consigned to the limbo of incurability.

One of the ablest surgeons of my acquaintance, in former years, had got his *materia medica* simplified to calomel and opium, and I am not sure if by this time even these have not been thrown overboard. It seems to be the surgeon's idiosyncrasy. Now I shall ever stand before the tribunal of this Association for the freest and most unfettered expression of opinion and judgment. Standing each "*on his own leather*," and given the privilege of combating what we regard as heterodox, crude, or baneful, there is nothing whereof to complain.

But I submit, Mr. President, that it is hardly the fair thing for one like the essayist of the evening, who almost wholly affects surgery, and devotes himself so zealously to his favorite branch, especially as he chances to be the Editor of the local *Medical Journal* of this part of the State, and who, as such, may, in a manner be regarded as the exponent of the views of the profession, as to our resources in the treatment of diseases; I submit, I repeat, if it is quite fair or commendable for one in his position to so boldly and frequently attack and attempt to bring into disrepute some of our resources so universally accepted as valuable and in many cases indispensable, as the essayist has done this eveuing, and as many of us think he is rather prone to do in his editorial capacity.

Of course, upon those of us who, from long and careful observation, have settled the question of their comparative value beyond the reach of carping, these inculcations will fall harmless. But how different with the inexperienced in practice, with whom the *Medical Journal*, and especially its editorial department, is apt to be regarded as supreme authority, or as a court of ultimate appeal?

Will the Doctor regard it as too personal if I say that in my judgment the tendency of such teachings from one in his position, is little, if anything, short of *pernicious*, upon many of the profession, and as such is to be deprecated? The truth is, I fear my friend must be regarded as well nigh heretical, certainly skeptical, in matters distinctively medical. Or, in other words, that the surgeon's *idiosyncrasy* is getting to be alarmingly developed in his case! And with the utmost deference to his intention and motive, will he allow me in all kindness to give expression to what is very often said, that he is apt to make quite too free with—to cut quite too deep and indiscriminate into, our cherished resources for the treatment of disease?

A word as to the question of the benefits of local, superficial, suppurative discharges, spontaneous or artificial, in the treatment of certain chronic maladies, whether cerebral, thoracic or spinal. If they have not in many cases proved of eminent service in either wholly arresting, or greatly postponing fatal diseases, and if the suspension or abatement of such discharges has not frequently precipitated a fatal result, the records of medicine must be regarded as irredeemably stupid in tracing the relation of cause and effect. If these effects and results have not been well established—proven beyond the possibility of cavil, it would seem entirely pertinent to question if *anything* is settled and certain in the therapeutics of medicine. If *this* is a matter of doubt, we may as well wipe out all testimony from observations and begin anew.

Dr. Trowbridge said, I think that Dr. Miner has been too sweeping in his assertions in regard to counter-irritants. It is true that our best medical men cannot always explain the action of remedies; although they may be fully satisfied, both from reason and experience, that their remedies produce the desired effect. Recollect many cases that occurred while I was in practice in which counter-irritants were of undoubted value. In 1849 saw a lady of this city who was taken with cholera, and suffered severely from that disease. After the subsidence of the cholera a persistent diarrhœa supervened. On recommendation of Dr. Pratt, with whom I saw the patient, a blister large enough to cover the whole abdomen was applied. As soon as the blister filled, the diarrhœa was cured. Blisters have been carelessly and indiscriminately used, but when carefully and properly used they are beneficial.

Dr. Cronyn observed, that he was a little disappointed in finding a difference in the title and matter of the paper read; that he came prepared to hear of some new method of tying an artery, treating an aneurism, or healing an old ulcer; instead of which the learned author had made an onslaught on time-honored medical remedies. It seemed to him that the paper involved the question so thoroughly discussed in England a few years ago, in which there were two extreme parties, to one of whom the author too evidently belongs, the one as will be remembered insisting that there was a decided change of type in disease, and consequently a change in its treatment, the antiphlogistic management of former days giving place to an opposite course in modern times, the other asserting with equal force that there was clearly a change in the therapeutical management of disease, that it was owing to a more comprehensive physiology and a better appreciation of pathological changes and undoubted advancement in diagnosis. To the former class he would connect the author, as a dose of opium to relieve pain, and stimulants to sustain failing strength, completes his *armamentaria medicorum*, ignoring the value recently attached to the use of blisters in rheumatic arthretis; also in the treatment of gonorrhœa, the actual cautery in painful and long standing sinovetis of knee-joint, especially, etc., etc. The fact was, as gentlemen exclusively directed their attention to the specialty of surgery, they became skeptics in the value of medical appliances, whose action was not as palpable as that of the knife in an amputation, and while the paper had unquestionable merit in itself, it would be wiser and more useful for the editor of the *Buffalo*

*Journal* to advocate the safer middle course between the two extremes referred to, so that his younger readers would have the advantage of learning how to discriminate between those rules, that sound pathology taught, and those changes that may occur in certain cycles of time in disease, and requiring at each time a difference in management, therapeutically and otherwise.

Dr. Lothrop thought the discussion had wandered away from the question, viz: the merits of counter-irritation. He would like to say a few words in support of the view presented by Dr. Miner, if he could. If Dr. M. meant to declare that in no case was counter-irritation of benefit, he should be obliged to differ from him, for he thought that it was often attended with most decided benefit. But if he meant only to say that it was sometimes needlessly employed, and that in many cases most unnecessary suffering, if not positive injury, was inflicted upon patients by its use, he could give him a most hearty assent.

The argument from experience was one which would cut both ways. For nothing was better shown than that remedies which were once deemed essential in acute diseases, and proved so seemingly by experience, are now by experience found to be unnecessary, if not absolutely injurious. But he was not yet prepared to allow that in this matter of counter irritation the experience of the profession from the earliest times, had been wholly fallacious. He believed that it was in proper cases productive of great benefit. He would, however, say that if the good effect of blisters were mainly due, as stated by Dr. Cronyn, to the absorption of cantharidin, it would answer the purpose quite as well, if given by the mouth. It appeared to him that the paper would have been more appropriately named, if styled the effects of counter-irritation.

Dr. Miner remarked, that he did not desire in any way to defend the paper—preferred to leave the objections to it unanswered. All that had been said, had been fully anticipated—was according to the current of opinion with physicians, and on that account, mainly, he had selected it as a topic for remark, hoping to attract more careful attention to the subject.

In regard to the title of the paper, he said: it had been handed to the Secretary, in full, and that the remedies had been called "surgical," in respect to the universal usage of authors.

Dr. Cronyn remarked, that the specimens presented at the last meeting of the Association, by Dr. Gay, and supposed by that gentleman to be gall-stones, proved on examination to be composed of oleum olivæ, saponified and mixed with a small amount of fæcal matter, shaped to resemble gall-stone in size and appearance, by the actiou of the intestine. Dr. C., as a result of his examination, felt himself warranted in denying the specific action of olive oil in favoring the discharge of gall-stones. It seemed to him rather to be true, that olive oil, given copiously, coincidently with pain in the neighborhood of the gall ducts—by changes in consistence and form, produced by the chemical and mechanical action of the intestine—would be discharged in such a shape as to be easily mistaken, upon a hasty inspection, for gall-stones.

Adjourned. T. M. Johnson, M. D., Sec'y.

